# Elements of Materia Medica and Therapeutics

**Published:** 1845-12

**Authors:** 


### REVIEWS.

---

Art. III.—*Elements of Materia Medica and Therapeutics.*—By John P. Harrison, M.D., Professor of Materia Medica and Therapeutics in the Medical College of Ohio. Vol. II. Philadelphia: Thomas, Coperthwaite & Co. Cincinnati: Desilver & Burr. 1845. 8vo. pp. 619.

We resume our notice of this work, the second volume of which came from the press a few weeks ago. The most highly elaborated chapter in this volume is the first, which treats of bloodletting. The author has brought together a great number of opinions, speculations and facts, relative to this curative process, affording the student an opportunity of seeing how discordant have been the views entertained of its value, and of the circumstances under which it should be practised. Celsus, we learn, "calls bloodletting the *optimum remedium;*" and Galen was not only "much addicted to the use of this powerful evacuant," but "transcended the points of its practical application laid down by his great master, Hippocrates." In all times it has been a favorite remedy with the profession; few physicians of any note have absolutely proscribed it, but some have gone so far as to interdict its use under all circumstances. Lest we should be suspected of having allusion to living characters we quote from Dr. Harrison, who informs us that—

"Van Helmont, upon very absurd hypothetical grounds, denounced this valuable means of cure. Chemical figments of disease so possessed his cultivated mind, that every sound deduction of observation was displaced by the erring light of an erroneous theory. Burserius trod, in servile imitation, in the steps of his predecessor, in opposing the claims of this remedial measure. From a groundless apprehension that concoction might be interrupted from an abatement of the 'brisk febrile motion, and degree of heat,' proper to that end, they eagerly traduced the beneficial tendency of bleeding in febrile and inflammatory attacks."

Concerning the mode of drawing blood by cups, our author has a suggestion which may be new to some readers; he proposes a sharp razor and a tumbler as substitutes for the more elaborate and costly apparatus commonly in use, and the preference, we, have no doubt, would generally be wise, for the common scarificator is apt to be so dull that the operator attempts in vain to incise the skin with it, where the parts are yielding, as, for example, over the abdomen. The razor, in such cases, would not fail. But we do not agree with our author that the preference of many practitioners for leeching, over the other method of local bleeding, is "extravagant." Circumstances there are, unquestionably, which render leeches ineligible, but under many other circumstances, which will occur to the mind of the reader, no other process of depleting can be adopted with so much comfort and advantage. It is unfortunate that they cannot be obtained by the majority of practitioners in the country; but wherever they are accessible we have found that they are in high repute with the sick, as well as their medical attendants.

The criteria by which we are to judge of the expediency of taking blood in disease, Professor H. lays down as five: "the pulse; the pain; disturbed functions; appearances exhibited by the blood; and tolerance of loss of blood." The question of abstracting the vital fluid is one, he says correctly, of great magnitude, and consistently with that view of the subject he devotes nearly two hundred pages to its consideration. He prefaces his observations on the several points just referred to by the following remarks:

"Bloodletting is an agent of great compass and efficiency in the cure of human maladies. It is our chief remedy in the severer forms of inflammatory seizure. Its power is familiar to the surgeon, to the accoucheur, and to the physician. The general practitioner, who combines in his own personal labors the duties of the surgeon, midwife, and physician, goes armed on all occasions with the appropriate instruments for drawing blood, and has recourse to them with a freedom and boldness which show that his reliance on this great therapeutic resource is of no doubtful character."

On the first head of his subject—the pulse—Dr. H. has collected a good deal that is curious in medical opinion and experience. Among other things of the sort, he alludes to the wish of Dr. Rush, that "the Chinese custom of prescribing from feeling the pulse only, without seeing or conversing with the patient, were imposed upon all physicians," which he pronounces "quite ultraism, upon a point in which we should exercise great calmness and reserve of judgment." Having got through with the literature of the subject, he goes on next to consider the physiology of the pulse, into which, however, we shall not follow him.

"What are the pathological states of the pulse?" This is the important question which comes next in order, and his division of the pulse is as follows:

"1. A full, slow, vigorous stroke of the artery.
"2. A full, tense, and frequent pulse.
"3. A hard, incompressible, quick pulse, feeling like a tightly drawn cord.
"4. A soft, open, compressible pulse.
"5. A small, rapid, and weak pulse.
"6. A creeping, soft pulse."

Upon the several varieties of the pulse as guides to the abstraction of blood, our author gives judicious cautions to the young practitioner, insisting, with entire correctness, that the pulse by itself cannot be regarded "as a nosometer." It may mislead in diseases of the heart, in gastritis, meningitis, rheumatism, and oppressed states of the system generally—indicating depletion where the remedy is not proper, and seeming to forbid it where it is called for. All that affects the pulse, therefore, must be attentively considered by the physician at the bed-side. The posture of the patient must be regarded, the state of his mind, the time of day, the age, sex, temperament of the subject, the seat and character of his disease, and the nature of the remedies previously given; all of which is pointed out under the head of "*directions for feeling the pulse.*"

The next indication is *pain;* but this may consist with opposite states of the system, and must be attentively considered before we decide upon the lancet as the means of removing it. Our author's views on this point are thus expressed:

"Plethora and anemia, congestion and bloodlessness, accumulation and decrease of action, may excite pain. The harmonious relation existing in a normal condition of the body, between the nervous and vascular systems, general and capillary, being broken by an essential departure from the quantum of blood, evinced either by increased or diminished distention of the sanguiferous tubes, pain will often be the result. Pain is most apt to arise when this relation, and reciprocative play, of the nervous and vascular systems are suddenly broken up, or disturbed. Thus a rapid nutrition, or a great loss of blood will excite pain in the head, which may portend serious encephalic disorder."

*Disturbed function as indicative of the propriety of blood-letting.* We have examples of this in the delirium attendant upon disease of the brain, in the painful respiration symptomatic of pleurisy and pneumonia, and in the convulsions of children originating in various causes. There is truth in the assertion of the author that—

"Deranged functions, when fairly interpreted by the lights reflected from the other phenomena of morbid action, will essentially conduce to a just inference as regards the expediency of vascular depletion."

The next indication—*the appearances of the blood*—our author discusses at considerable length. With Heberden he holds, that as diseases have not their seat in the blood, so its sensible qualities have but little relation to them. In like manner, also, he rejects the idea, that the blood possesses vitality. He says—

"To talk of the distinct organization of the blood is gratuitous mysticism in anatomy. To assert that the blood exhibits the same vital phenomena, and is possessed of the same endowments of sensibility and irritability as the solids, is transcendental, and staggers all belief."

The true conception of the blood we take to be that it is the pabulum of the system—the fibrin and albumen, of which the various organs and tissues are constructed. As to the mind of our author, so to us there is something gratuitous in the assertion, that it is endowed with the vital properties of the solid parts of the body.

The author is certainly safe in rejecting the appearance of the blood as a certain or safe index of propriety of bloodletting. Pathologists are busily engaged in the investigation of the blood as affected by disease, and results, we may hope, will be brought out affording us accurate and applicable tests for determining the character of the affection and the kind of remedies demanded; but all our knowledge on that subject at present is rather curious than practical. Other tests besides the buffy coat of the blood must be brought to our aid in all questions as to the expediency of bleeding. Dr. Harrison dwells upon this subject, and his conclusions are sanctioned by the experience of the profession.

*Tolerance of loss of blood* is the last index of our author to the propriety of further bleeding, and on this point, as on the last, his views are such as practical men must generally approve. He condemns the practice of bleeding to syncope, except in cases of urgent necessity, and adduces a host of arguments against, it any one of which might well give the inexperienced physician pause.

*Circumstances which modify the effects of bloodletting;* these are indicated in the next section and will receive the attention of the student; but we pass over them to section V, which treats of the *practical applications of bloodletting.* The author cites the following cases in illustration of the large amount of blood which may be drawn with safety to the patient, in certain forms of disease:

"Extraordinary quantities of blood have been detracted from patients affected with cerebral inflammation. Ninety ounces of blood were taken at one time from Dr. Dewees by Dr. Physick, in severe cerebral disease. Dr. Dewees drew 120 ounces within five or six hours, and 20 more on the following day, from a patient affected with puerperal convulsions. In another patient, similarly affected, he took 80 ounces at one bleeding. These patients all recovered."

This whole subject—the lancet in inflammatory affections—is well treated, and the student will find in the admonitions of Dr. H. safe rules for managing a most formidable class of diseases. He says, truly in regard to bleeding in rheumatism—

"Professor Bouillard errs in the excess to which he carries sanguinous depletion in acute rheumatism. We are to remember the liability of this affection to a transference to the pericardium, and reflect that blood may be drained away, until, as Dr. Barlow remarks, the body is blanched and the crassamentum reduced to a tithe of its just proportions, yet febrile action will continue, inflammation be unsubdued, and the blood drawn be still buffed and cupped."

Among the maladies not curable by the loss of blood, our author mentions tetanus, and, in the following language, hydrophobia:

"Hydrophobia has been subjected to the entire ordeal of pharmaceutic powers, from bleeding to *deliquium animi*, down to the infinitessimal doses of the *ars homœopathia*—through mercury, arsenic, prussic acid, &c.—and yet it remains a standing testimony to the fallacy of all human contrivance to subdue its unmitigable mastery over life. Prevention, by early excision of the bitten part, is the only expedient that promises aught in this terrible malady."

In regard to the employment of the lancet in fevers, we must content ourselves with quoting the circumstances mentioned by our author as indicating its necessity; they are:

"1. High vascular action, denoted by great thirst, urgent heat of the skin, and full strong pulse. 2. great determination to the brain, evidenced by pain, throbbing, and exaltation of sensibility in the sense of sight, or hearing, or by delirium, convulsions, or coma. 3. Intense gastric irritability, denoted by incessant vomiting. 4. Obstinate constipation. 5. Hemorrhage from the stomach, lungs, or bowels. 6. Suppression of urinary secretion. 7. Threatened abortion in pregnant women. 8. Tenderness upon carefully conducted abdominal pressure. 9. Intense pain in the back. 10. Red tongue, with epigastric oppression."

The sentiment of Prof. H. is a just one, that the only safe indications of cure in fever are the symptoms. If we suffer preconceived notions to shackle or mislead us, the symptoms will address themselves to us in vain. We have then eyes, but see not; ears, but hear not; as earnestly declared by our author—

"No abuses are so pregnant of evil as those trenched in the strong holds of an irrefutable theory. Let the young physician be instructed by the errors of his predecessors on the blandishments of any universal scheme of doctrine, which looks only at half of the difficulties of a question, and which obliges its defender to put himself in an attitude of resistance to every fresh accession of information on certain debated matters."

To the consideration of bloodletting in diseases, succeeds a valuable section on the *morbid effects of the loss of blood*, with which the chapter appropriately closes. The importance of the topic commends it to the attention of every practitioner. Much discrimination is necessary in the use of the lancet, and the question, how far the symptoms are incident to the disease, or consequent upon the depletion, is one by which the physician is sometimes seriously perplexed.

There are auxiliaries to bleeding, and substitutes for it, in febrile and inflammatory diseases; these are enumerated by Prof. H., and are:

"A strict regulation of diet, perfect rest in a horizontal position, the protracted application of cold to the surface of the body, the use of cold drinks, the exhibition of nauseants and emetics, purgatives, and of mercury."

Of cold water he says—

"The affusion of cold water to the general surface in fevers accompanied by high arterial action, indicated by quickened pulse and hot, dry skin, stands conspicuous among the successful measures to subdue the febrile orgasm.

"Cold water as a drink is a valuable auxiliary to bloodletting in fever, and various inflammatory affections. Especially where gastritis portends, ice water is a most efficient auxiliary in the reduction of the general excitement, as well as gastric hyperæmia. An error is sometimes committed in the excessive quantity of cold water allowed in such cases. If the stomach becomes inordinately filled with water, oppression and much distress, with an aggravation of the constitutional disturbance, as well as local difficulty, will ensue."

We should say, as a general rule, let the patient have cold water *ad libitum;* if his stomach be oppressed by it, he will vomit, and thus, if not positively useful, it will do no harm. Still there are cases where this indulgence might be pernicious; experience must decide under all circumstances.

At length we reach the second chapter, which treats of *emetics.* This is introduced by a dissertation on the physiology of vomiting. We agree with Prof. H. that emetics do not act by being absorbed into the circulation, but by their peculiar impression on the nerves of the stomach. How the contents of the stomach are ejected—whether by the contraction of that organ itself, or by the contraction of other parts upon it—is still a matter of dispute. Magendie has attempted to show by experiment, that the stomach is inert in the process of vomiting; Maingault has performed other experiments to prove that vomiting may take place after the division of the diaphragm and abdominal muscles. Upon this our author has these words:

"Now, let us not be misled by these experimental vivisections. In our view they demonstrate too much, and therefore only prove what may be done under the unnatural conditions present. To vomit without a stomach, or to vomit without the aid of the diphragm and abdominal muscles, may answer for a poor lacerated, mutilated dog, but to adduce such experiments as proofs of what occurs as a physiological function in man is neither evidential of sound induction, nor are such data to be relied on as furnishing the axiomata of a safe medical creed."

The management of emetics, their practical employment, the diseases in which applicable, and their combination with tonics, stimulants, narcotics and other medicines, together with a description of individual emetics, make the subjects treated in this chapter—a chapter abounding in just views, and details of practice in which the student will find valuable instruction. If any one should be disposed to object to the details as being unnecessarily minute, it should be remembered that this work was written for students, and was meant to be elementary. The following passage, descriptive of the fate of antimonial remedies, is in the style of the "*Triumphant Chariot of Antimony.*" Says the author, referring to the popularity conferred upon antimony by this immortal work of Basil Valentine—

"But from this noon-tide of reputation, the remedy was doomed, for a while, to sink, beneath the proscription of the Supreme Council of Paris, which fulminated against it a furious edict in 1566, and in obedience to which Vandal objurgation, Besnier was expelled the Faculty of Medicine, for having had the temerity, contrary to the Galenical or exclusive vegetable doctors of the day, to administer it to a patient. For some years, antimony lay concealed beneath the opprobrium thus cast upon it, till, slowly emerging from its obscure and proscribed state, it gradually rose to a high position among the leading articles of Materia Medica. Cullen of Scotland, and Fordyce of England, contributed much in advancing the reputation of antimonial preparations, and the more recent announcements of the Italian school of contra-stimulus, with the less speculative statements of French and English practitioners, have trimphantly advanced antimony to a proud and impregnable place of renown and power."

Under the head of *the morbid effects of tartar emetic*—the author relates the following case, which shows that the remedy is by no means destitute of danger under all circumstances:

"Miss L. H., a young lady of mobile, excitable, nervous system, 18 years of age, was taken with bilious remittent fever, whilst her mind was in a state of impassioned excitement, from family difficulties connected with a love affair of her own. Four grains of tartar emetic dissolved in eight spoonfuls of water were given in separate quantities, of a tablespoonful each, every ten minutes. Copious vomiting took place—alarming prostration ensued—the pulse sunk rapidly—incessant jactitation with cold clammy skin came on, and in despite of the most diffusible stimulants, she died on the third day after the administration of the medicine."

The author gives the Italian practice with tartar emetic, which he appears not to have found available. Has any American physician? He says—

"Rasori's method, so much eulogized and, we may add, so impracticable in the cases we have had to treat, was, after one or more bleedings, or without this evacuation, according to circumstances, to give twelve grains of tartarized antimony twice during the twenty-four hours. If the disease had made considerable progress, he began with twenty or thirty grains, increasing the dose daily till one drachm, or even several drachms, were taken in the course of twenty-four hours. Of 832 cases of pneumonia thus treated, only 173 died. Laennec, adopting the practice of Rasori, restricted the quantity of the remedy to a comparatively limited extent. After bleeding, he prescribed one grain every two hours, increasing it to two, or two and a half grains, at the same intervals. At first it generally puked and purged, but these effects soon passed off, and the *tolerance* was established often within twenty-four hours from the time of its first administration. It was occasionally necessary to give aperients to keep the bowels open. It was discovered by him that the remedy was most efficacious when it did not produce any sensible evacuation. Pneumonia had been treated according to Laennec, by Riverius and Stoll with tartar emetic as a vomit; given every day, or every other day. And Dumangin, physician to La Charite, scarcely ever combined bloodletting with it, and yet his success in the treatment of pneumonia was equal to that of Corvisart in that affection, though by him vascular depletion was freely employed."

His conclusions respecting the employment of tartar emetic in pulmonary affections, are thus stated:

"1. That the liberal use of the lancet should precede, in severe forms of pneumonia, the exhibition of the remedy.

"2. That the employment of tartar emetic should not supercede local bloodletting in the disease.

"3. That the remedy is adapted to the first and second stages of pneumonia, in which engorgement and red hepatization obtain.

"4. That from one grain to four grains every two hours is as much as our patients will commonly bear.

"5. That counter-irrritation by blisters may be advantageously used, after the more urgent inflammatory symptoms are subdued, during the time that the tartar emetic is administered.

"6. That in protracted, as well as in typhoid attacks, the constitutional agency of mercury must be associated with the remedy.

"7. That the tolerance of the stomach in bearing large doses of the medicine does not altogether arise from the character of the attack, but is in part dependent upon a repetition of the dose, habituating the gastric mucous tissue to its action: also in part dependent on the vehicle in which it is given.

"8. That the anti-inflammatory power of the remedy is distinct from its nauseant and evacuant modes of operation, and partakes of a species of rapid, energetic alterative influence.

"9. That whenever decided symptoms of gastritis, or enteritis, are present, the remedy must be either altogether laid aside, or employed with much caution.

"10. And that a combination of one or two grains of calomel, ten grains of nitrate of potash, and one grain of tartar emetic, is the best form of its exhibition, in very dangerous attacks of pulmonic phlegmasia. Also, in subdued forms of the disease a union of calomel, tartar emetic, and digitalis, or opium, is the most eligible mode of exhibiting our antimonial."

The chapter on *cathartics* is brief, in comparison with the foregoing, or with that which treats of bloodletting. This topic is disposed of in fifty pages; and, in that space, besides laying down precise rules for the guidance of the young practitioner, Prof. H. contrives to cite a multitude of authorities in reference to purgatives. Nor does he forget to speak of the abuse of this class of medicines. But we have not room to follow him over all these subjects, much less to engage in the discussion of themes so fruitful in controversy.

Under the head of *incitants* the author groups tonics, antispasmodics, and astringents; and we have an account of acetate of lead and alcohol in the same chapter.

The author's remarks on the mode of administering the preparations of cinchona will be read with interest:

"*Modes of exhibiting cinchona, and the disulphate of quinia.* The bark in substance was formerly our almost exclusive reliance in the arrest of intermittent fever. But, however managed, it was an arduous undertaking to get many patients to take enough of it for the attainment of the object. It was mixed with wine, or porter, and thus introduced into the stomach; or it was united with Virginia snake root and ginger, and a hot infusion made of it; or it was thrown up the rectum; or it was quilted into a flannel shirt, and the patient's body surrounded by it thus applied. The best two forms of its exhibition were its combination with snake root and ginger, as already stated, and its union with pulverized Virginia snake root and carbonate of potash. When prepared in the last mentioned mode, it was given in molasses or ginger syrup. Very often, to a warm infusion of bark and snake root, after it was cold, was added dilute sulphuric acid. After all our best devised plans of its administration, it was a hard task, very often, to master an intermittent by the bark in substance, for, in addition to the difficulty of its exhibition, was the very frequent adulteration of the drug.

"Amidst all this perplexity and vexation, the great discovery of the di-sulphate of quinine burst upon us. Feelingly do we speak on this point, from the fact of our practising in a miasmatic district of country, in which intermittent fever was exceedingly rife, and often very unmanageable. The science of modern chemistry, amid its great and glorious achievements, perhaps never conferred a richer boon on practical medicine, than by the discovery of this preparation. And now, familiarized as we are to its use, it behooves us with renewed attention, to study well its curative powers, that we may, with increased skill, more successfully combat those morbid states which demand its interference.

"The di-sulphate of quinine is endowed with the following properties:

"1. Pre-eminently it possesses an anti-periodic power.

"2. It is decidedly tonic.

"3. It is an excitant to the nervous and vascular systems.

"4. In an inferior degree it is astringent.

"5. It possesses a febrifuge quality.

"6. It is slightly diaphoretic.

"The basis of the great reputation of the bark was laid in the unrivalled mastery which it was found to exercise over intermittent fever.

"After the cinchona had acquired a conspicuous position among the curative means in which mankind reposed confidence, and had been sold for its weight in silver, as a remedy in fever and ague, then so common and so fatal in various parts of Europe, it fell into an almost total neglect, until its popularity was revived by a circumstance which brought forward the article from its concealment and neglect. Allusion is had to the fact that Louis XIV purchased of Sir Robert Talbor, an Englishman, a secret remedy employed by Talbor with great success in intermittent fever. This secret remedy proved to be the bark, which from this period stood forward as one of the great commanding agents employed in the practice of medicine.

"No article, nor combination of articles, of the Materia Medica, can for a moment stand a comparison with bark as an antiperiodic remedy. This attribute of the cinchona is distinctive, pre-eminent, and energetic. It is to a very limited extent participated in by other vegetable tonics, and among our mineral agents, arsenic is the only remedy that produces similar results in periodical attacks.

"This characteristic property of the bark is, without abatement of virtue, transferred to the di-sulphate of quinine. In doses varying from one grain to half a drachm, the di-sulphate asserts a similar dominion to the cinchona in arresting febrile and other seizures, which recur in regular paroxysms.

"The most successful method of securing the exercise of this antiperiodic property, is to give the di-sulphate of quinine in a large dose two hours anterior to the expected onset of the intermittent. Our usual dose is not less than ten grains, and somtimes we exhibit at one time as high as thirty. Where much debility obtains, we often prescribe two grain doses, every two hours, during the entire apyrexia, and then one hour before the anticipated seizure, order five or ten grains.

"The pilular form is the preferable mode of its exhibition. In this form morphine may be combined with it, in cases where there is irritability of the stomach, or in which diarrhœa exists. In phlogistic states of the system we often combine tartarised antimony, or ipecacuanha with the di-sulphate."

Dr. Harrison speaks well of *iron.* He quotes Dr. Elliotson and others for its virtues in chorea and other diseases, but might have said much more of it as a remedy in chronic intermittents. Of the tincture of the muriate he says:

"The muriated tincture of iron is one of our best chalybeates. Endued with tonic and astringent properties to an extent not equalled by any other ferruginous preparation, it is likewise specially capacitated to act on the genito-urinary apparatus. As a tonic, we have found it very effective in apepsia, or want of peptic energy. Where pyrosis is the predominant symptom, no tonic has better succeeded in our hands. In slight attacks of chorea it acts most propitiously. In anemia, our main reliance has usually been the muriated tincture. If torpor of bowels is present, the tincture of aloes may be advantageously combined with it. As an internal astringent, the remedy has proved productive of good in diarrhœa, but is apt, in full doses, like alum, to induce slight catharsis, though in small quanties it exercises an astringent action on the digestive tube. As an external styptic it has been applied, with benefit, to indolent ulcers.

"Its capability of exerting a special medicinal influence on the uterus can scarcely be questioned by any one who has made a faithful application of it to cases of retained and suppressed menstruation, occurring in weakened habits. Like the sesqui-oxide it always communicates to the alvine discharges a dark color."

*Restoration of the secretions* is the heading of the chapter succeeding, and among the articles calculated to fulfil this indication we find enumerated, diaphoretics, baths, diuretics, and antilithics, expectorants, emmenagogues, and anthelmintics. Then follows a chapter on the *anodyne*, or *narcotic indication of cure*, and the volume closes with a consideration of the *revulsive indication of cure.*

We have thus attempted to present an outline of the subjects with which this volume is taken up, and some account of the doctrines of the author on a few points. The reader will not have failed to discover, that in many respects the work has stronger claims to originality than any system of materia medica which our country claims the honor of having produced. In a former article we indicated some particulars in which it struck us that it was susceptible of improvement. We shall add nothing more to what was said on that occasion, but only remark, that we shall be glad to find in future editions of the work, if new editions should be called for, that the author has corrected the errors of doctrine and style to which the press has pretty generally adverted.

